# Dietary calcium requirements of broilers fed a conventional corn-soybean meal diet from 1 to 21 days of age

**DOI:** 10.1186/s40104-021-00652-5

**Published:** 2022-02-03

**Authors:** Shiping Bai, Yunfeng Yang, Xuelian Ma, Xiudong Liao, Runlian Wang, Liyang Zhang, Sufen Li, Xugang Luo, Lin Lu

**Affiliations:** 1 Mineral Nutrition Research Division, State Key Laboratory of Animal Nutrition, Institute of Animal Science, Chinese Academy of Agricultural Sciences, Beijing, 100193 China; 2 Poultry Mineral Nutrition Laboratory, College of Animal Science and Technology, Yangzhou University, Yangzhou, 225000 China; 3 Institute of Animal Nutrition, Sichuan Agricultural University, Chengdu, 611130 Sichuan China; 4 Department of Animal Science, Guangdong Ocean University, Zhanjiang, 524088 China; 5 Department of Animal Science, Hebei Normal University of Science and Technology, Qinhuangdao, 066004 China

**Keywords:** Bone characteristic, Broiler, Calcium, Gene expression, Requirement

## Abstract

**Background:**

The current calcium (Ca) recommendation for broilers is primarily based on studies conducted more than 30 years ago with birds of markedly different productive potentials from those which exist today. And the response indicators in these studies are mainly growth performance and bone ash percentage. Therefore, the present study was carried out to investigate the effect of dietary Ca level on growth performance, serum parameters, bone characteristics and Ca metabolism-related gene expressions, so as to estimate dietary Ca requirements of broilers fed a conventional corn-soybean meal diet from 1 to 21 days of age.

**Methods:**

A total of 420 1-day-old Arbor Acres male broilers were randomly assigned to 1 of 7 treatments with 6 replicates (10 birds per cage) and fed the corn-soybean meal diets containing 0.60%, 0.70%, 0.80%, 0.90%, 1.00%, 1.10% or 1.20% Ca for 21 days. Each diet contained the constant non-phytate phosphorus content of about 0.39%.

**Results:**

The average daily gain decreased linearly (*P* < 0.001) as dietary Ca level increased. The serum and tibia alkaline phosphatase (ALP) activities, tibia bone mineral density (BMD), middle toe BMD, tibia ash percentage, tibia breaking strength, and tibia ALP protein expression level were affected (*P* < 0.05) by dietary Ca level, and showed significant quadratic responses (*P* < 0.02) to dietary Ca levels. The estimates of dietary Ca requirements were 0.80 to 1.00% based on the best fitted broken-line or quadratic models (*P* < 0.03) of the above serum and bone parameters, respectively.

**Conclusions:**

The results from the present study indicate that the Ca requirements would be about 0.60% to obtain the best growth rate, and 1.00% to meet all of the Ca metabolisms and bone development of broilers fed a conventional corn-soybean meal diet from 1 to 21 days of age.

## Background

Calcium (Ca) is an essential mineral element, and plays an important role in many biological processes, such as enzyme activation, intracellular signaling, acid-base balance and bone mineralization [1]. As about 99% of Ca is stored in skeleton as hydroxyapatite, Ca is crucial to the bone development of broilers [2]. Skeletal abnormalities attract great concern in poultry industry as they cause not only production problems but also considerable animal welfare issues. The deficiencies in content or improper ratios of Ca and phosphorus (P) were usually considered as main nutritional reasons leading to greater incidences of leg abnormalities and huge economic loss [3].

The current Ca recommendations for broilers by NRC (1994) [4] were 1.00%, 0.90%, and 0.80% for the starter (1 to 21 d), grower (22 to 42 d), and finisher (43 to 56 d) phases, respectively. However, these recommendations are based on studies conducted in 1960s and 1980s [5, 6], and the response indicators in these studies are mainly growth performance and bone ash percentage. The modern broilers differ very much from those before 1980s in the growth rate, feed conversion efficiency, carcass quality and bone characteristics [7–9]. The feed efficiency of modern broiler chickens has been improved [7], and it is assumed that the feed intake has decreased, thus the modern broiler chickens have produced the same adequate bone traits with reduced Ca. Therefore, the Ca requirements of modern fast-growing broilers might be different from those of broiler strains reared several decades ago. However, as Ca appears to be less threatening to the environment than P [10], and the sources of Ca, namely limestone or oyster shell flour, are cheaper than other mineral sources, the determination of Ca requirements receives little attention [11]. It has also been reported that modern broilers have a high capacity to adapt P or Ca deficiency [12]. Nevertheless, the high level of dietary Ca may reduce the energy value of the diet and interferes with the availability of other minerals [13]. Decreasing the amount of dietary Ca may improve performance, but this should not be at the expense of increased leg problems [14]. Furthermore, the starter phase (1 to 21 d) is a critical period for bone development of broilers. Thus it is necessary to re-evaluate dietary Ca requirements of broilers fed a conventional corn-soybean meal diet from 1 to 21 days of age.

Chicken bone mineral density (BMD) or content (BMC) determined by dual-emission X-ray absorptiometry (DEXA) was shown to be highly correlated with bone ash percentage, which could well reflect the Ca nutritional status [15]. Furthermore, the enzymes and proteins involved in Ca utilization were found sensitive to the dietary Ca levels in animals, especially alkaline phosphatase (ALP), osteocalcin (OC), osteoprotegerin (OPG), and bone morphogenetic protein-2 (BMP-2) were considered as good biological markers of bone metabolisms [16–19]. However, the above mentioned indicators have never been used to estimate dietary Ca requirements of chickens. We hypothesized that BMD, BMC and mRNA or protein expression levels of ALP, OC, OPG and BMP-2 in bone might be new sensitive indices to evaluate dietary Ca requirements of broilers fed a corn-soybean meal diet, and dietary Ca requirements of broilers from 1 to 21 days of age might be different from the current NRC Ca requirement (1.00%).

Therefore, the objective of the current study was to investigate the impact of dietary Ca levels on growth performance, serum parameters, bone characteristics and Ca metabolism-related gene expressions of broilers, so as to choose sensitive indices to evaluate dietary Ca requirements of broilers fed a corn-soybean meal diet from 1 to 21 days of age.

## Materials and methods

All experimental procedures were approved by the Animal Management Committee of the Institute of Animal Science, Chinese Academy of Agricultural Sciences (IAS-CAAS, Beijing, China), and performed in accordance with the guidelines. Ethical approval on animal survival was given by the animal ethics committee of IAS-CAAS. The ARRIVE guidelines were followed for reporting animal research [20].

### Experimental design, animals and diets

A total of 420-day-old Arbor Acres male broiler chicks (Huadu Broiler Breeding Corp) with similar body weights were randomly allotted to 1 of 7 treatments with 6 replicate cages of 10 birds per cage in a completely randomized design, and housed in an electrically heated, thermostatically controlled room with fibreglass feeders, waterers and stainless-steel cages coated with plastics for 21 d. They were maintained on a 24-h constant light schedule and allowed ad libitum access to experimental diets and tap water. The basal corn-soybean meal diet (Table 1) was formulated to meet or exceed the requirements [4, 21] of broilers for all other nutrients except for Ca and P. Dietary Ca levels for 7 treatments were calculated to be 0.60%, 0.70%, 0.80%, 0.90%, 1.00%, 1.10% and 1.20% Ca, respectively. The requirement value of dietary non-phytate P (NPP) recommended by the current NRC [4] is 0.45%. In the present study, each diet contained the constant NPP content of about 0.39% based on the results of our previous study [22]. A single large batch of the basal diet without limestone, was mixed at first, and then divided into 7 sublots according to the experimental treatment. Each sublot was mixed with limestone (containing 38.7% Ca by analysis) or fine sand [23, 24]. The washed fine sand containing no detectable Ca and P was used to maintain the same weight of each treatment diet. The dietary Ca levels by analysis on an as-fed basis were 0.59%, 0.70%, 0.80%, 0.88%, 0.98%, 1.08% and 1.18%, respectively. All diets were fed in mash form. At the end of the experiment, after fasting for 12-h, broiler weight and feed intake were recorded for each replicate cage and corrected for mortality to calculate average daily gain (ADG), average daily feed intake (ADFI) and feed: gain (FCR) from 1 to 21 days of age.

**Table.1:** Composition and nutrient levels of the basal diet for broilers from 1 to 21 days of age (as-fed basis)

Item	Contents
Ingredients, %	
Corn	52.97
Soybean meal	37.51
Soybean oil	5.00
CaHPO_4_^a^	1.72
Limestone^a^	0.24
Salt^a^	0.30
DL-Met^a^	0.31
Micronutrents^b^	0.33
Sand	1.62
Nutrient levels, %	
ME, MJ/kg	12.69
Crude protein ^c^	21.62
Lysine	1.12
Methionine	0.59
Threonine	0.80
Tryptophane	0.23
Methionine + Cystine	0.90
Non-phytate phosphorus	0.39
Calcium^c^	0.59

^a^ Feed grade

^b^ Provided per kilogram of diet: VA, 15,000 IU; VD_3_, 4500 IU; VE, 24 IU; VK_3_, 3 mg; VB_1_, 3 mg; VB_2_, 9.6 mg; VB_6_, 3 mg;VB_12_, 0.018 mg; Pantothenic acid calcium, 15 mg; Niacin, 39 mg; Folic acid, 1.5 mg; Biotin, 0.15 mg; Choline, 700 mg; Zn (ZnSO_4_.7H_2_O) 60 mg; Cu (CuSO_4_•5H_2_O) 8 mg; Mn (MnSO_4_•H_2_O) 110 mg; Fe (FeSO_4_•7H_2_O) 40 mg; I (KI) 0.35 mg; Se((Na_2_SeO_3_) 0.35 mg

^c^ Determined values based on triplicate measurements, and the others were calculated values

### Sample collections and preparations

At the end of the experiment, chicks in each cage were individually weighed after fasting for 12-h, and 12 chicks (2 chicks per cage) from each treatment were selected according to average body weight of each cage, respectively. Blood samples were promptly obtained using wing vein puncture, and then centrifuged to harvest serum and stored at − 20 °C for analyses of ALP activity and Ca or inorganic P contents. Birds were then killed by cervical dislocation. The left tibia and middle toe were collected and stored at − 20 °C for analyses of bone characteristics. The right tibia was snap-frozen in liquid N_2_ and then stored at − 80 °C for assays of ALP, BMP-2, OC or OPG mRNA and protein expression levels. To reduce individual biological variation, samples from two chickens in each replicate cage were pooled into one sample in equal ratios before analyses, and thus there were a total of 6 replicate samples for each treatment.

### Serum parameters

Serum inorganic P was determined by vanadate-molybdate method [25]. Serum Ca and ALP activities in serum and tibia were measured using commercial assay kits (Nanjing Jiancheng Bioengineering Institute, Nanjing, China).

### Bone characteristics

The frozen tibia and middle toe were thawed at room temperature for 2 h, and BMC and BMD were determined by DEXA using the case of small animal model (Lunar_iDXA; GE healthcare, Madison, WI, USA). After the scan, tibia and middle toe were immediately rebagged and frozen until further analyses. The tibia bone strength was determined using a texture analyser (TA.XT plus; Stable Micro Systems, London, UK). The tibia was put on a fulcrum point with 50 mm apart. Loading point was located in the midpoint of fulcrum points. The value of breaking force was determined by the shear test at a speed of 5 mm/min with a 50 kg loading cell until fracture occurred. The ultimate breaking force of the tibia was indirectly obtained, according to the load v. deformation curve recorded by computer. Following this testing, the tibia and middle toe were dried at 105 °C for 24 h and defatted with fresh diethyl ether for 48 h, and then dried at 105 °C for 12 h to determine bone weight. The dried and defatted tibia and middle toe were ashed in muffle furnace at 550 °C for 24 h to measure bone ash weight and calculate bone ash percentage.

### Diet and bone ash analyses

Samples of diets and bone ash were ground in a laboratory mill to pass through a 0.5 mm screen. The crude protein level in the basal diet was analyzed according to the Kjeldahl method [26]. The Ca concentrations in diets and bone ash were determined by inductively coupled plasma spectroscopy (Model IRIS Intrepid II; Thermo Jarrell Ash, Waltham, MA, USA). Total P concentrations in diets and bone ash were determined with a spectrophotometer [27]. Validations of Ca and total P analyses were conducted concurrently using soybean powder (GBW10013; National Institute of Standards and Technology, Beijing, China) as a standard reference material.

### RNA extraction and quantitative RT-PCR assays

The total RNA was isolated from the tibia by using TRIZOL reagent (Invitrogen Life Technologies, Carlsbad, CA, USA) according to the manufacturer’s instructions. The concentration of total RNA was estimated by measuring its optical density at 260 and 280 nm with a spectrophotometer (ND-100; NanoDrop Technologies, Wilmington, DE, USA). A 500 ng of total RNA was reversely transcribed into cDNA using PrimeScript™ RT Master Mix Kit (TaKaRa Bio Inc., Otsu, Japan) according to the manufacturer’s instructions. The cDNA was used as templates for real-time quantitative PCR amplification using SYBR green master mix (Applied Biosystems, Foster City, CA, USA) on the ABI 7500 Real-Time PCR machine following the manufacturer’s guidelines. The gene-specific primers for *ALP*, *BMP-2*, *OPG*, *OC*, β-actin and glyceraldehyde-3-phosphate dehydrogenase (*GAPDH*) are shown in Table 2. Internal reference genes, both β-actin and *GAPDH*, were constant across the dietary treatment groups, and thus their geometric mean could be used to normalize the expression of the targeted gene [28]. Relative gene expression was calculated using the ^2-ΔΔCt^ method [29].

**Table.2:** Primer sequences for real-time PCR amplification

Gene	GenBank ID	Primer sequences	Length, pb
β-actin	NM_205518.1	F: 5′-ACCTGAGCGCAAGTACTCTGTCT-3′	152
		R: 5′-CATCGTACTCCTGCTTGCTGAT-3′	
*GAPDH*	K01458	F: 5′-CTTTGGCATTGTGGAGGGTC-3′	128
		R: 5′-ACGCTGGGATGATGTTCTGG-3′	
*ALP*	NM_205360.1	F: 5′-GGAGAAGGACCCCGAATACTG-3′	300
		R: 5′-TTGACGCCGCAGAGGTAAG-3′	
*OPG*	XM_015283019.2	F: 5′-ATCTCAGTCAAGTGGAGCATC-3′	186
		R: 5′-GTTCCAGTCTTCAGCGTAGTA-3′	
*OC*	NM_205387.3	F: 5′-TGCTCGCAGTGCTAAAGCCTTCAT-3′	143
		R: 5′-TCAGCTCACACACCTCTCGTT-3′	
*BMP-2*	NM_204358.1	F: 5′-CCAACACCGTGTGCAGCTT-3′	136
		R: 5′-TGGAGTTCAGCTGAGGTGACAGA-3′	

^a^ Abbreviations: *GAPDH* glyceraldehyde-3-phosphate dehydrogenase, *ALP* alkaline phosphatase, *OPG* osteoprotegerin, *OC* osteocalcin, *BMP-2* bone morphogenetic protein-2

### Western blotting assays

After ground in liquid N_2_, the tibia samples were homogenized in 0.5 mL of ice-cold radio immunoprecipitation assay lysis buffer (Beyotime Biotechnology, Haimen, China) supplemented with 5 μL of protease inhibitor (BioTool, Houston, TX, USA). The homogenate was centrifuged at 10,000 × *g* for 10 min at 4 °C, and the supernatant was collected for total protein determination using a BCA Protein Assay Kit (Beyotime Biotechnology, Shanghai, China). The extracted protein (20 μg) was subjected to electrophoresis on a 10% SDS-PAGE gel, and then electrotransferred onto the polyvinylidene fluoride membranes (Merck-Millipore, Munich, Germany). After the transfer, membranes were blocked for 1 h at room temperature in a blocking buffer with 5% non-fat milk, and then incubated overnight at 4 °C with the following primary antibodies: ALP (A6866; ABclonal, Wuhan, China), OPG (A135204; ABclonal, Wuhan, China), OC (A18241; ABclonal, Wuhan, China), BMP-2 (A0231; ABclonal, Wuhan, China) and β-tubulin (HX1829; Huaxingbio, Beijing, China). After washing, the membranes were incubated with the secondary antibody of goat anti-rabbit (HX2028; Huaxingbio, Beijing, China) or goat anti-mouse (HX2113; Huaxingbio, Beijing, China) for 1 h at room temperature. The signals were recorded with automatic chemiluminescence imaging analyzer (5200 Multi; Tanon, Shanghai, China) by Chemistar ECL Western Blotting Substrate (180–501; Tanon, Shanghai, China). Data were presented as the ratio of ALP, OPG, OC or BMP-2 protein band intensity to β-tubulin protein band intensity.

### Statistical analyses

Data from the present study were subjected to one-way ANOVA using the general linear model procedure of SAS (v. 9.2, SAS Inst. Inc., Cary, NC, USA), and differences among means were tested by the least significant difference method. The replicate cage of 9 to 10 chickens for growth performance or two chickens for other indicators served as the experimental unit for all statistical analyses. Data from mortality were transformed to arcsine for analysis. Orthogonal comparisons were applied for linear and quadratic responses of dependent variables to independent variables. Regression analyses of broken-line, quadratic and asymptotic models were performed, and the best fitted models between responsive criteria and dietary Ca level concentrations were used to determine dietary Ca requirements (the break point from broken-line model or the maximum response from quadratic model) of broilers [30]. The level of statistical significance was set at *P* ≤ 0.05.

## Results

### Growth performance and mortality

Dietary Ca level affected (*P <* 0.005) body weight on d 21 and ADG of broilers from 1 to 21 days of age, but did not affect (*P >* 0.10) ADFI, FCR and mortality (Table 3). The body weight on d 21 and ADG decreased linearly (*P* < 0.0001) as dietary Ca level increased. Broilers had the highest ADG at dietary Ca level of 0.59%. Furthermore, all broilers did not show leg abnormality during the whole experimental period.

**Table.3:** Effect of dietary calcium (Ca) level on the growth performance of broilers during 1 to 21 d of age^1^

Analyzed dietary Ca level, %	Body weight on d 21, g	ADFI, g/d	ADG, g/d	FCR, g/g	Mortality^2^, %
0.59	825^a^	46.32	37.13^a^	1.25	1.67
0.70	793^b^	44.90	35.64^b^	1.26	1.67
0.80	781^bc^	44.43	35.09^bc^	1.27	3.33
0.88	792^b^	44.98	35.58^b^	1.26	0.00
0.98	789^b^	44.83	35.46^bc^	1.27	0.00
1.09	768^bc^	43.83	34.46^bc^	1.27	0.00
1.18	757^c^	43.43	33.94^c^	1.28	0.00
SEM	10.8	1.668	0.096	0.029	0.017
*P*-value					
Ca level	0.004	0.11	0.004	0.71	0.33
Linear	< 0.0001	–	< 0.0001	–	–
Quadratic	0.72	–	0.72	–	–

^1^Data represented the means of 6 replicates (*n* = 6)

^2^Mortality was based on analysis after anti-sine transform

^a-c^Means within a column with unlike superscript letters were significantly different (*P* < 0.05)

### Serum parameters and ALP activity in the tibia

Dietary Ca level did not affect (*P* > 0.05) serum Ca and inorganic P contents, and Ca to inorganic P ratio of broilers (Table 4), but affected (*P* < 0.05) ALP activities in serum and tibia. As dietary Ca level increased, the ALP activity in serum increased linearly (*P =* 0.0003) and quadratically (*P* = 0.002), and it in tibia decreased quadratically (*P* = 0.005). The minimum ALP activity in serum was observed at 0.98% Ca level, and that in tibia at 0.80% Ca level.

**Table.4:** Effect of dietary calcium (Ca) level on serum parameters and tibia ALP activities of broilers on d 21^1^

Analyzed dietary Ca level, %	Serum Ca content, mg/100 mL	Serum inorganic P, mg/100 mL	Serum Ca:inorganic P ratio	Serum ALP Activity, King unit/100 mL	Tibia ALP Activity, King unit/g prot
0.59	8.63	6.22	1.39	312^cd^	384^a^
0.70	8.65	7.09	1.23	380^bc^	350^ab^
0.80	8.21	6.78	1.22	304^cd^	271^c^
0.88	8.50	7.14	1.19	345^bcd^	305^bc^
0.98	8.95	6.92	1.30	294^d^	340^ab^
1.09	8.63	6.81	1.27	396^b^	348^ab^
1.18	8.76	6.36	1.39	486^a^	355^ab^
SEM	0.553	0.534	0.133	51.9	57.1
*P*-value					
Ca level	0.43	0.06	0.08	< 0.0001	0.04
Linear	–	–	–	0.0003	0.82
Quadratic	–	–	–	0.002	0.005

^1^Data represented the means of 6 replicates (*n* = 6)

^2^Abbreviation: *ALP* alkaline phosphatase

^a-d^Means within a column with unlike superscript letters were significantly different (*P* < 0.05)

### Tibia characteristics

Dietary Ca level did not affect (*P* > 0.39) tibia ash Ca or P contents and Ca to P ratio, but affected (*P* < 0.03) the tibia ash percentage, BMD, BMC and breaking strength (Table 5). As dietary Ca level increased, tibia ash percentage and BMD increased quadratically (*P* < 0.004), but tibia BMC increased linearly (*P* = 0.012). In addition, tibia breaking strength increased linearly (*P* = 0.001) and quadratically (*P* = 0.012) with increasing dietary Ca levels. Tibia ash percentage or breaking strength reached a plateau, and tibia BMD reached the highest point at 0.88% Ca.

**Table.5:** Effect of dietary calcium (Ca) level on tibia bone parameters of broilers on d 21^1^

Analyzed dietary Ca level, %	Tibia ash percentage, %	Tibia ash Ca content, %	Tibia ash P, %	Tibia ash Ca: P ratio	Tibia BMD, mg/cm^2^	Tibia BMC, g	Tibia breaking strength, N
0.59	49.3^bc^	35.1	17.8	1.96	136^bc^	0.88^b^	98^c^
0.70	48.7^c^	35.3	17.9	1.97	133^c^	0.82^b^	109^bc^
0.80	50.2^ab^	35.6	17.9	1.98	146^b^	0.98^ab^	129^ab^
0.88	50.9^a^	35.8	18.0	1.99	158^a^	1.00^ab^	140^a^
0.98	50.6^ab^	35.3	17.9	1.97	145^b^	1.02^ab^	134^ab^
1.09	50.5^ab^	35.6	17.9	1.99	141^bc^	1.10^a^	131^ab^
1.18	49.4^abc^	35.6	17.6	2.03	142^bc^	0.95^b^	132^ab^
SEM	1.17	0.79	0.34	0.052	8.0	0.105	19.7
*P*-value							
Ca level	0.004	0.73	0.40	0.45	0.0003	0.027	0.008
Linear	0.07	–	–	–	0.11	0.012	0.001
Quadratic	0.003	–	–	–	0.0006	0.052	0.012

^1^Data represented the means of 6 replicates (*n* = 6)

^2^Abbreviations: *BMC* Bone mineral content, *BMD* bone mineral density

^a-c^Means within a column with unlike superscript letters were significantly different (*P* < 0.05)

### Middle toe characteristics

Dietary Ca level did not influence (*P* > 0.11) the middle toe ash percentage, P content, Ca to P ratio and BMC, but influenced (*P* < 0.009) its ash Ca content and BMD (Table 6). The middle toe ash Ca content increased linearly (*P* < 0.0001), but its BMD increased quadratically (*P* < 0.0001) as dietary Ca level increased. The highest middle toe BMD was observed visually at 0.88% dietary Ca level.

**Table.6:** Effect of dietary calcium (Ca) level on middle toe bone parameters of broilers on d 21^1^

Analyzed dietary Ca level, %	Toe ash percentage, %	Toe ash Ca, %	Toe ash P, %	Toe ash Ca: P ratio	Toe BMD, mg/cm^2^	Toe BMC, g
0.59	41.6	36.9^c^	17.99	2.04	45^c^	0.13
0.70	41.2	37.2^bc^	18.14	2.06	51^bc^	0.15
0.80	41.91	37.6^abc^	18.22	2.10	55^ab^	0.20
0.88	42.1	37.6^abc^	18.51	2.07	56^a^	0.18
0.98	41.9	37.8^ab^	18.24	2.06	53^ab^	0.18
1.09	42.3	37.9^a^	18.07	2.08	46^c^	0.15
1.18	41.2	38.1^a^	17.99	2.09	46^c^	0.17
SEM	0.215	0.530	0.324	0.053	4.5	0.046
*P*-value						
Ca level	0.20	0.008	0.12	0.52	< 0.0001	0.18
Linear	–	< 0.0001	–	–	0.37	–
Quadratic	–	0.55	–	–	< 0.0001	–

^1^ Data represented the means of 6 replicates (*n* = 6)

^2^ Abbreviations: *BMC* bone mineral content, *BMD* bone mineral density

^a-c^ Means within a column with unlike superscript letters were significantly different (*P* < 0.05)

### mRNA levels of ca metabolism-related genes in the tibia

Dietary Ca level affected (*P* < 0.03) tibia *OC* and *BMP-2* mRNA expression levels, but did not affect (*P* > 0.30) tibia *ALP* and *OPG* mRNA expression levels (Table 7). As dietary Ca level increased, both *OC* and *BMP-2* mRNA levels decreased linearly (*P* < 0.03).

**Table.7:** Effect of dietary calcium (Ca) level on ALP, OPG, OC and BMP-2 mRNA and protein expression levels in the tibia of broilers on d 21^1^

Analyzed dietary Ca level, %	*ALP* mRNA, RQ^2^	*OPG* mRNA, RQ^2^	*OC* mRNA, RQ^2^	*BMP-2* mRNA, RQ^2^	ALP protein, RQ^3^	OPG protein, RQ^3^	OC protein, RQ^3^	BMP-2 protein, RQ^3^
0.59	1.11	0.98	1.69^a^	0.91^ab^	0.89^c^	1.14	1.75	1.03
0.70	1.06	1.19	1.24^ab^	1.07^a^	1.03^abc^	1.03	1.62	0.81
0.80	0.84	0.97	0.85^bc^	0.72^bc^	1.17^ab^	0.98	1.67	0.79
0.88	0.89	0.85	1.00^bc^	0.88^abc^	1.19^a^	0.91	1.65	0.73
0.98	1.00	1.00	1.00^bc^	1.00^ab^	1.06^abc^	0.88	1.88	0.79
1.09	0.87	0.74	0.65^c^	0.79^abc^	0.95^bc^	1.03	1.58	0.85
1.18	0.75	0.79	0.66^c^	0.66^c^	1.03^abc^	1.01	1.43	0.89
SEM	0.107	0.113	0.147	0.092	0.069	0.089	0.134	0.081
*P*-value								
Ca level	0.37	0.31	0.002	0.02	0.04	0.49	0.37	0.25
Linear	–	–	< 0.0001	0.02	0.75	–	–	–
Quadratic	–	–	0.11	0.24	0.006	–	–	–

^1^Data represented the means of 6 replicates (*n* = 6)

^2^The mRNA levels were calculated as the relative quantity (RQ) of the target gene mRNA to the geometric mean of β-actin and glyceraldehyde-3-phosphate dehydrogenase mRNA, RQ = 2^-ΔΔCt^ (Ct, threshold cycle)

^3^The protein levels were calculated as the relative quantity (RQ) of the target gene protein to the glyceraldehyde-3-phosphate dehydrogenase protein

^4^Abbreviations: *ALP* alkaline phosphatase, *BMP-2*, morphogenetic protein-2, *OC* osteocalcin, *OPG* osteoprotegerin

^a-c^Means within a column with unlike superscript letters were significantly different (*P* < 0.04)

### Protein expression levels of ca metabolism-related genes in the tibia

Dietary Ca level affected (*P* = 0.04) tibia ALP protein expression levels, but did not affect (*P* > 0.24) tibia OPG, OC and BMP-2 protein expression levels (Table 7). The tibia ALP protein expression level increased quadratically (*P* = 0.006) as dietary Ca level increased. The highest tibia ALP protein expression level was observed visually at 0.88% dietary Ca level.

### Estimations of dietary ca requirements of broilers

Results of dietary Ca requirements of broilers as estimated by the non-linear regression analyses are shown in Table 8. The results indicate that serum and tibia ALP activities, tibia ash percentage, tibia breaking strength, tibia and middle toe BMD, and tibia ALP protein expression level were suitable criteria for evaluating dietary Ca requirements of broilers. Based on the best fitted broken-line or quadratic models of the above criteria, optimal dietary Ca levels were estimated to be 1.00%, 0.80%, 0.93%, 0.88%, 0.93%, 0.91% and 0.90% for broilers fed a conventional corn-soybean meal diet from 1 to 21 days of age. However, broilers had the highest ADG at dietary Ca level of 0.59%. Therefore, in general, the Ca requirement would be about 0.60% to obtain the best growth rate and 1.00% to meet all of the Ca metabolisms and bone development of broilers fed a conventional corn-soybean meal diet from 1 to 21 days of age.

**Table.8:** Estimations of dietary calcium (Ca) requirements of broilers from 1 to 21 days of age based on the best fitted broken-line or quadratic models

Dependent variable	Regression equation^a^	R^2^	*P*-value	Dietary Ca requirements, %
Serum ALP activity	*Y*_*1*_ = 79.44–79.44*X* (0.59 ≤ *X* ≤ 1.00) *Y*_*2*_ = − 670.1 + 999.9*X* (1.00 ≤ *X* ≤ 1.18)	0.49	< 0.0001	1.00
Tibia ash percentage	*Y*_*1*_ = 44.895 + 6.5766*X* (0.59 ≤ *X* ≤ 0.93) *Y*_*2*_ = −55.9373 - 5.3306*X* (0.93 ≤ *X* ≤ 1.18)	0.29	0.005	0.93
Tibia ALP activity	*Y*_*1*_ = 668.4286–473.9648*X* (0.59 ≤* X* ≤ 0.80) *Y*_*2*_ = 134.7242 + 193.1789*X* (0.80 ≤ *X* ≤ 1.18)	0.27	0.007	0.80
Tibia breaking strength	*Y*_*1*_ = 11.0110 + 145.1498*X* (0.59 ≤ *X* ≤ 0.88) *Y*_*2*_ = 162.1505–27.2524*X* (0.88 ≤ *X* ≤ 1.18)	0.37	0.0005	0.88
Tibia BMD	*Y* = 0.0285 + 0.2592*X* - 0.1400*X*^2^	0.24	0.005	0.93
Tibia ALP protein expression level	*Y* = −0.6983 + 4.0791*X* - 2.2713*X*^2^	0.14	0.021	0.90
Middle toe BMD	*Y *= −0.0341 + 0.2024*X* - 0.1153*X*^2^	0.46	< 0.0001	0.91

^a^Regression equations based on the analyzed Ca concentrations (%) in the diets

^b^Abbreviations: *ALP* alkaline phosphatase, *BMD* bone mineral density

## Discussion

Our hypotheses that BMD, BMC and mRNA or protein expression levels of ALP, OC, OPG and BMP-2 in bone might be new sensitive criteria to evaluate dietary Ca requirements of broilers fed a corn-soybean meal diet, and dietary Ca requirements of broilers from 1 to 21 days of age might be different from the current NRC Ca requirement (1.00%) have been partially supported by the results of the current study. The current study indicated that tibia and middle toe BMD, serum and tibia ALP activities, and tibia ALP protein level were new sensitive criteria to evaluate dietary Ca requirements of broilers, and the Ca requirement would be about 0.6% to obtain the best growth rate and 1.00% to meet all of the Ca metabolisms and bone development of broilers fed a conventional corn-soybean meal diet from 1 to 21 days of age. These findings could better characterize requirements and meet the growth, bone development and Ca metabolic functions of broilers.

In earlier studies, growth performance was often used to assess Ca requirements of broilers [6, 13]. In order to maximize the growth performance of broilers, using diets with 0.6% Ca becomes more widespread [2]. As birds possess specific Ca appetite [31], they get adapted to low Ca diets by increasing absorption and utilization efficiency, which decreases excretion of the restricted nutrients [12] and elevates plasma 1,25-dihydroxyvitamin D_3_ (1,25(OH)_2_D_3_) concentration and duodenal calbindin concentrations [32, 33]. The current study showed that the increase of dietary Ca level from 0.70 to 1.18% had a negative impact on growth rate of broilers from 1 to 21 days. Similar results were reported by Walk et al. [9], indicating that the increase of dietary Ca level depressed the weight gain and feed intake at a 0.32% dietary NPP level. The possible reason for the negative effect of high-Ca diet on broiler’s growth rate might be due to that relatively high Ca reduced P availability [34], leading to the formation of extremely insoluble Ca-phytate complexes and severe P deficiency. In addition, higher dietary Ca could increase intestinal pH, which reduced the absorptivity of minerals [35]. Hamdi et al. [13] found that broilers achieved their greatest weight gain with 0.70% dietary Ca and 0.38% dietary NPP. Additionally, some researchers recommended relatively lower levels of dietary Ca (0.60 to 0.65%) for broilers according to weight gain and feed intake [36, 37], which is similar to the results from the present study. However, Valable et al. [38] and Wilkinson et al. [39] reported that the reduction of dietary Ca did not affect the growth performance of broilers. Different breeds and growth phases of broilers might account for the discrepancy of the above results.

The Ca and P contents in serum and bone have been considered as good parameters to reflect the nutritional status of Ca in broilers [40, 41]. However, the current study showed that dietary Ca level had no effect on serum and tibia Ca, P content and Ca to P ratio, indicating that these parameters were not suitable to estimate the Ca requirements. Some researchers reported similar results [42, 43]. Hurwitz et al. [44] explained that modern broilers had good ability to keep serum Ca and P contents in narrow range regardless of the varying dietary Ca levels mainly because of the oscillations regulation system, in which parathyroid hormone, calcitonin and 1,25(OH)_2_D_3_ play an important role. In addition, the Ca content in middle toe ash increased linearly with increasing dietary Ca levels, indicating that middle toe ash Ca is not a useful marker for the assessment of Ca requirement of broilers.

Bone characteristics, such as bone ash percentage and breaking strength, have been traditional criteria to evaluate bone mineralization in broilers [22]. The NRC Ca recommendations for broilers were mainly based on maximizing bone ash. According to the results from the current study, the Ca requirements were 0.93% and 0.88% based on tibia ash percentage and breaking strength, respectively. And these two criteria increased quadratically as dietary Ca increased, which is in line with the results reported by Bar et al. [10]. However, some previous studies showed that the Ca requirement (1.3%) based on bone ash contents of 21-d-old broilers was higher than the current NRC Ca requirement (1.00%) [6, 45], which might be due to the high dietary P levels (from 0.7 to 0.75%) in these studies. Shafey [46] reported that increasing dietary Ca enhanced the breaking strength of cockerel tibia. However, Onyango et al. [15] did not observe the same phenomenon, and thought that the high variability in breaking strength contributed to the lack of statistical significance. Actually, several factors can affect breaking strength, such as cross head speed, handling of bones before testing and measurement technique [15, 47, 48]. Some researchers determined BMC and BMD of broilers using DEXA [49, 50]. The DEXA technology provided a rapid and noninvasive advantage measurement of bone mineralization compared with traditional measurement. Yan et al. [12] found that BMC and BMD were good indicators for Ca nutrition estimation. Onyango et al. [15] and Valable et al. [38] reported that BMC and BMD showed a linear and quadratic increase with the increase of dietary Ca level, which is similar to the results from the present experiment. The optimal Ca level was estimated to be 0.93% and 0.91% based upon tibia and middle toe BMD, which was close to that (0.93%) estimated by the tibia ash percentage. Onyango et al. [15] showed that there was a high correlation between bone ash and BMC or BMD. The above results indicate that the requirement for maximizing bone development was greater than that for the best growth performance of broilers, which was further confirmed by Bar et al. [10]. It might be viable to reduce the dietary Ca level in order to maximize the growth performance of broilers, however, it should not be at the expense of bone health.

Alkaline phosphatase is a ubiquitous enzyme that catalyzes the hydrolysis of phosphate monoesters [51], and can be used as a general indicator of skeletal development [52]. The increase of ALP activity is usually associated with inadequate supply of Ca or P and poor bone mineralization [53, 54]. Xia et al. [55] found that serum ALP activity was sensitive to the dietary Ca, and can be used to evaluate the dietary Ca requirements for laying Longyan shelducks. The current study showed that both serum and tibia ALP activities changed quadratically as dietary Ca increased, and the Ca requirements to obtain the minimum ALP activities in serum and tibia were 1.00% and 0.80%, respectively. Similarly, Hurwitz and Griminger [56] reported that serum ALP activity decreased as dietary Ca increased, reaching a minimum in the range of probable Ca requirement, and could be used as an indicator to evaluate Ca adequacy in growing chicks. In the present study, the tibia ALP protein expression level increased quadratically as dietary Ca increased, and the Ca requirement obtained from this indicator was 0.90%, while the tibia *ALP* mRNA expression level was not affected by dietary Ca. Mehra et al. [57] explained that changes in protein levels were not directly related to changes in mRNA levels, because of the complexity of transcription, translation and posttranslational modification. Another consideration might be the time course of the experiment. The tibia *ALP* mRNA may have been upregulated early in the study in response to dietary treatments, and by the end of the study, its expression levels may have returned to baseline values.

Bone metabolism includes bone formation and resorption. Markers of bone formation and resorption act as key determinants in the regulation of bone mass [17, 58]. Osteocalcin is the most abundant non-collagenous protein of bone matrix and plays an important role in the bone formation and Ca metabolism [59]. Osteoprotegerin is a secreted protein involved in the regulation of bone resorption [58, 60]. The decrease of *OPG* mRNA expression in the tibia led to the osteoporosis in broilers [61]. Jiang et al. [19] reported that the increase of dietary Ca level enhanced *OPG* mRNA and OC protein expression levels in the keel bone of hens. However, in the present study, dietary Ca levels did not affect *OPG* mRNA and OC protein expression levels in the tibia of broilers. The disparity may mainly result from the different animal species (previous hens vs. present broilers) and bone samples. The BMP-2, a potent osteogenic differentiation factor, is essential for osteoblast differentiation and bone formation [62]. Jia et al. [63] found that the mRNA and protein levels of BMP-2 increased with increasing Ca^2+^ concentrations in the primary renal tubular epithelial cells. A study with piglets demonstrated that the serum BMP-2 concentration exhibited a trend from rise to decline with increasing dietary Ca levels [64]. Similar tendency was observed for the tibia *BMP-2* mRNA levels as dietary Ca levels increased in the present study. In addition, the results from the present study showed that as dietary Ca levels increased, the tibia *OC* and *BMP-2* mRNA levels changed linearly, but not quadratically, indicating that these parameters are not suitable to estimate the requirements of Ca in broilers. We also found that the mRNA levels of *OC* and *BMP-2* in the tibia varied, while their protein levels did not change as dietary Ca levels increased, implying that the transcriptional change would often precede the translational change [60, 65].

The present study showed that the Ca requirement was 0.59% based on ADG. Similarly, Sebastian et al. [36] found that the optimum body weight, feed intake, and feed efficiency were obtained at 0.60% dietary Ca. We also found that the Ca requirements of broilers ranged from 0.8 to 1.00% based on tibia and middle toe characteristics, serum or tibia ALP activity, and tibia ALP protein expression level. In order to meet all of Ca metabolisms and bone development functions, dietary Ca requirement would be 1.00% for broilers fed a conventional corn–soybean meal diet from 1 to 21 days of age, which is in line with the current NRC Ca requirement. Previous reports suggested that the Ca requirement should be higher for skeletal development than for optimal growth [10, 66]. Our findings also suggest that the current NRC (1994) recommendation for Ca (1.00%) would be adequate for the optimum bone development but excessive for the best growth rate.

## Conclusions

The results from the present study indicate that 0.59% dietary Ca level was sufficient to obtain the best growth rate of broilers form 1 to 21 days of age. However, considering the serum and tibia ALP activities, tibia ash and breaking strength, tibia and middle toe BMD, and tibia ALP protein expression level, the Ca requirement of broilers would be 1.00% to support all of the Ca metabolisms and skeletal development, which is the same as the current NRC Ca requirement.

## Data Availability

The data are shown in the main manuscript.

## Abbreviations

ADFI: Average daily feed intake
ADG: Average daily gain
ALP: Alkaline phosphatase
BMC: Bone mineral concentration
BMD: Bone mineral density
BMP-2: Bone morphogenetic protein-2
Ca: Calcium
DEXA: Dual-emission X-ray absorptiometry
1,25(OH)_2_D_3_: 1,25-dihydroxyvitamin D_3_
GAPDH: Glyceraldehyde-3-phosphate dehydrogenase
IAS-CAAS: Institute of Animal Science, Chinese Academy of Agricultural Sciences
NPP: Non-phytate phosphorus
OC: Osteocalcin
OPG: Osteoprotegerin
P: Phosphorus
